# Local Anodic Oxidation of Graphene: The Role of Number of Layers, Load Force, and Substrate

**DOI:** 10.1021/acsomega.5c10137

**Published:** 2026-01-22

**Authors:** Jan Vymazal, Miroslav Bartošík, Martin Konečný, Jakub Piastek, Jindřich Mach, Linda Supalová, Ondřej Špaček, Tomáš Šikola

**Affiliations:** † Central European Institute of Technology − Brno University of Technology (CEITEC BUT), Purkyňova 123, 612 00 Brno, Czech Republic; ‡ Institute of Physical Engineering, Brno University of Technology, Technická 2, 616 69 Brno, Czech Republic; § Department of Physics and Materials Engineering, Faculty of Technology, Tomas Bata University in Zlín, Vavrečkova 5669, 760 01 Zlín, Czech Republic

## Abstract

Local anodic oxidation has become a convenient technique for fabricating graphene oxide nanostructures in fundamental research (e.g., nanoelectronics). The process is typically controlled by tip–sample voltage, scanning speed, relative humidity, and tip characteristics (e.g., tip radius). The role of other parameters, such as the number of layers, load force, and graphene-substrate adhesion, is discussed in this paper. It is shown by atomic force microscopy, Kelvin probe force microscopy, and Raman spectroscopy that the oxidation of graphene is achievable only under specific conditions: low pulling force and sufficiently strong adhesion of graphene to its substrate. Such conditions ensure the stability of graphene on the surface and the proper formation of the water meniscus, which serves as a source of oxidizing ions, resulting in a reproducible oxidation process. Failure to comply with these conditions may lead to the formation of structures other than oxides (e.g., removal of graphene or the formation of air/water cavities under graphene), which is also demonstrated.

## Graphical abstract

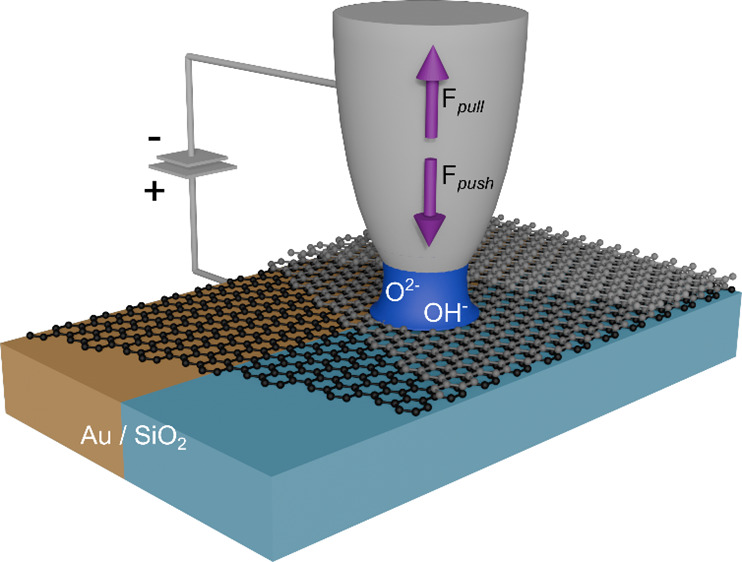

## 1 Introduction

Graphene belongs to the well-known two-dimensional materials, discovered at the beginning of this century. It is a crystal of carbon atoms packed into a honeycomb structure, possessing extraordinary electronic properties and characteristics, such as high charge carrier mobility, low electronic noise, sensitivity to adsorbents, biocompatibility, or the ability to be patterned by different lithographic methods. Thanks to them, it is a proper material for manufacturing a wide range of electronic nanodevices.^1–7^

Local anodic oxidation (LAO), conducted by atomic force microscopy (AFM), has become a convenient technique for designing prototypes of graphene nanoelectronics (creating isolating barriers of nanometer dimensions) or biosensors (binding functional groups with graphene oxide). Nevertheless, AFM LAO is a complex process of many parameters, including the relative humidity (RH) of the ambient environment (as water is the source of oxygen ions),^3,8,9^ the voltage applied between the AFM tip and the grounded graphene sample,^3,8,10,11^ the load or pulling force of the tip (excessive force might detach the graphene),^9^ the velocity of the tip,^3,9,12^ the thickness of the graphene layers (the monolayer behaves differently from a bilayer or multilayer) and the substrate (different substrates interact differently with the graphene).^3,7–12^

Some of these variables have already been studied extensively. A voltage threshold between −3.5 and −5 V was observed, while voltages below this value did not affect graphene.^3,8,10,11^ Relative humidity is also a crucial factor in LAO processing; the oxidation effect was not apparent under an RH threshold of approximately 30–40%.^3,8,9^ Specific dependence on the tip load force was not found.^9^ The tip velocity experiments showed that the LAO process occurs only at a lower speed of 40–100 nm/s, as higher velocity prevents the formation of a water meniscus.^3,9,12^

This paper examines the remaining parameters: load force, number of graphene layers, and role of the underlying substrate. While earlier studies did not explore how tip load force affects LAO results, this influence is highlighted here. Additionally, previous work used graphene layers without considering the effect of different layer numbers. This study systematically investigates this aspect through experiments and simulations, comparing the behavior of monolayers, bilayers, and multilayers. Furthermore, the article discusses how LAO processing varies with substrate type, comparing silicon dioxide and gold, and analyzing the impact of different adhesion forces between graphene and these substrates. It also demonstrates that Kelvin probe force microscopy (KPFM) and subsequent data analysis can offer insights into the relationship between oxidation and charge diffusion in graphene.

## 2 Methods

### 2.1 Sample Preparation

Graphene layers were mechanically exfoliated from a graphite crystal and transferred by an adhesive tape to squares (area of ∼1 cm^2^) of polydimethylsiloxane (PDMS). High-quality monolayer flakes (see Figure 1a) were identified using AFM (topography) and Raman spectroscopy. The I­(2D)/I­(G) intensity ratio of 2D and G Raman peaks assessed the graphene quality and thickness. It is known that high-quality graphene monolayers meet a ratio of about 2 to 3 (see Figure 1b).^3,13,14^ High-quality graphene was transferred onto an experimental platform equipped with gold electrodes (see Figure 1c) on a 285 nm thick nonconductive layer of silicon dioxide (SiO_2_), thermally grown on a silicon substrate. The graphene layers were placed on the SiO_2_ layer while partially covering a gold electrode, being electrically grounded through a copper wire attached to the electrode via a silver paste.

**Figure 1:**
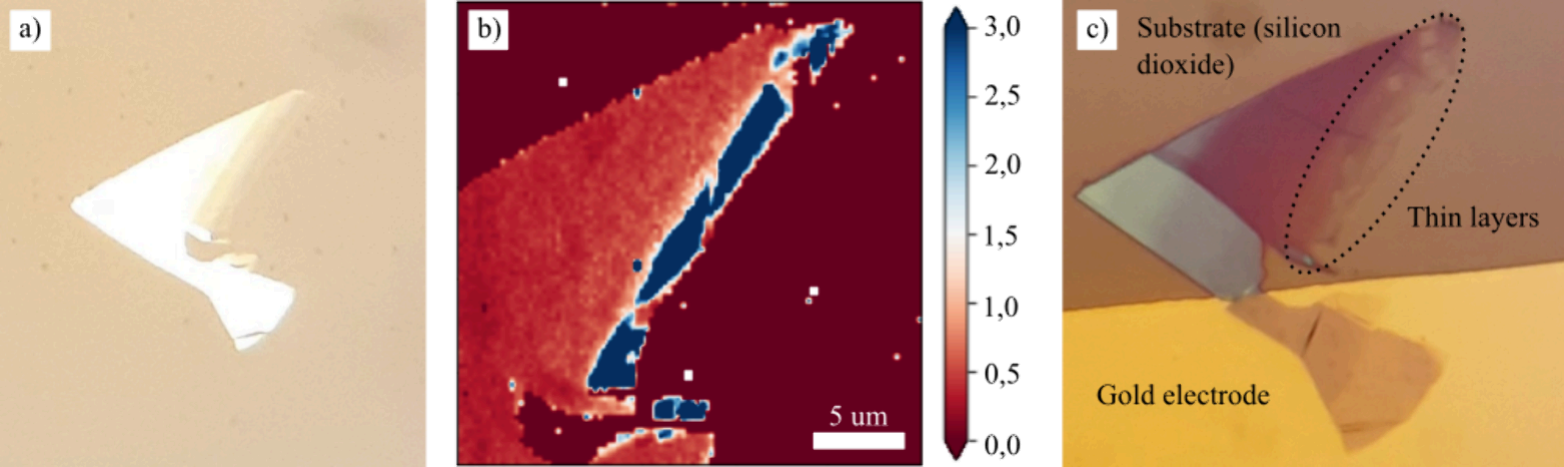
(a) Exfoliated graphene layer on PDMS (optical microscope). (b) Map of the I­(2D)/I­(G) ratio of Raman peak intensities. (c) Graphene layer; placed over the gold electrode and the silicon dioxide substrate. The thickness ranges from thin layers (marked) to thick ones (up to 50 nm), to study the influence of the number of graphene layers on the LAO process.

The transferred graphene layer, as shown in Figure 1, varies in thickness from a graphene monolayer to graphite (approximately 50 nm thick). Thus, it provides an environment to study the effect of different layer numbers on the oxidation process.

### 2.2 Experiments

The experimental sections aimed at an LAO study concerning the influence of (i) the number of layers, (ii) the loading force and (iii) the substrate, with the latter two being interrelated.

In the first section of experiments, the LAO conditions were as follows: relative humidity of 66 ± 2%, an oxidizing voltage of −9 V, a load force of 5–9 nN (during pulling, see Section 3.2), the tip–sample velocity of 1 μm/s, and a processed surface area of 1 μm^2^. The area was processed through 256 line scans, spaced approximately 3.9 nm apart. Due to the width of the line, which is in the range of tens of nanometres, each point within the processed area was affected multiple times. A conductive boron-doped diamond AFM tip DCP10 (NT-MDT) was utilized. An array of squares was created by LAO across graphene, with a thickness ranging from one to six layers.

Similar conditions were also used for the second and third sections of experiments marked above as (ii) and (iii). To study LAO for higher pushing and pulling forces, another tip, DCP20, with higher cantilever stiffness, was employed. That enabled load forces varying from about 10 nN in pulling mode to hundreds of nanonewtons in the pushing mode. These experiments were carried out on thicker graphene layers (3–5 layers). These layers were placed both on the gold electrodes and the SiO_2_ layer to compare the effects of different substrates.

In all the experimental sections, the resulting structures were characterized by AFM, Kelvin probe force microscopy (microscope NTEGRA II, NT-MDT Spectrum Instruments), and Raman spectroscopy (alpha300 R, Oxford Instruments WITec). KPFM measurements were repeated over several weeks to study the time evolution of the surface potential.

### 2.3 Simulations

The electric potential and field distribution between the AFM tip and graphene layers were calculated using the Maxwell equations for electrostatic problems in the COMSOL Multiphysics software. The geometry of the simulated configuration (in cylindrical symmetry) is depicted in Figure 2. The AFM tip is represented by a sphere of radius *R*, separated by the distance *h* from the graphene/graphite surface. In the simulation, graphene layers are represented by a stack of 0.3 nm thin layers of graphite (with relative permittivity ε_r_ = 12), separated by intercalated air (ε_r_ = 1) layers of the same thickness. The simulations were done for a graphene single layer, bilayer, four-layer and eight-layer, to demonstrate the influence of the graphene stack thickness.

**Figure 2:**
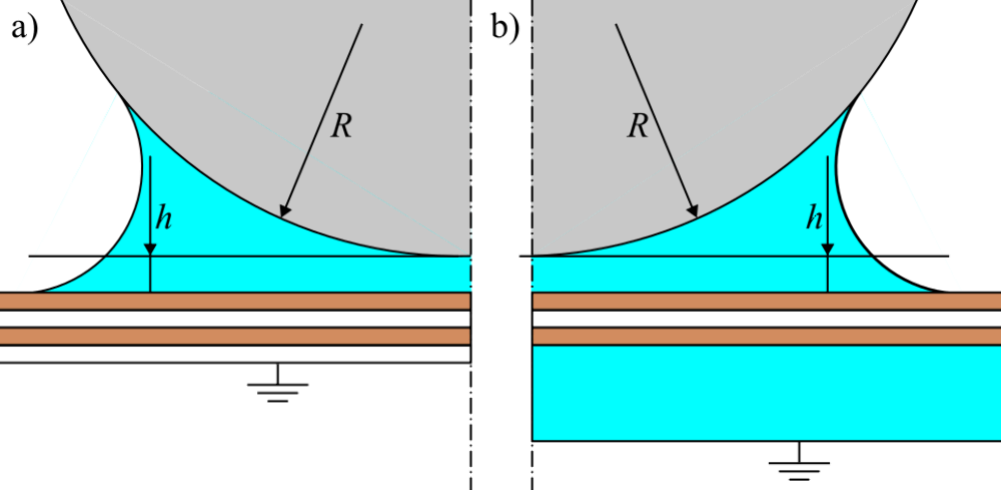
Geometry of the simulation: AFM tip (gray) approximated by a sphere of radius *R*, separated by the distance *h* from the stack of graphene layers (graphene layers in brown, air intercalated layers in white). An approximate shape of the water meniscus (cyan) was formed between the tip and the graphene. There were two cases of simulation: (a) a thin air layer and (b) a thick water layer under the graphene layers.

Further, two cases were considered. In the first case, air (ε_r_ = 1) was under the bottommost graphene layer (see Figure 2a). In the second case, water (ε_r_ = 81) was placed there (see Figure 2b). This case is based on previous studies, observing the water molecules intercalating between graphene sheets and their substrates, such as SiO_2_,^15,16^ MoS_2_,^17,18^ and mica.^19,20^ The intercalated water layer was included as 1.5 nm thick, based on topography measurements in this study, supported by corresponding results in the literature.^21^

The moisture of the air (relative humidity, RH) leads to the formation of a water meniscus between the tip and the sample. The meniscus shape was calculated using an approximate model using the Kelvin equation (see Supporting Information), supposing perfect wettability of the water (so the meniscus is tangential to both tip and graphene).^29,30^ The width of the meniscus grows with relative humidity and radius of the tip, while increasing distance *h* results in narrowing the meniscus. The relative permittivity of the meniscus is equal to that of water (ε_r_ = 81). An oxidizing voltage *U* was applied to the AFM tip, while the bottommost intercalated layer (air/water) was grounded.

## 3 Results

### 3.1 Number of Graphene Layers

Three square-like structures shown in Figure 3 were prepared using LAO on distinct numbers of graphene layers. The first structure almost fully occurred on a monolayer – the area marked I. On the other hand, the second structure spans over “layer steps” from two layers (marked II, right-bottom corner) to six layers (marked VI, left-top corner). The third structure is located entirely on the six layers. Making structures over many graphene layers enables us to make relevant conclusions on their influence on the LAO process.

**Figure 3:**
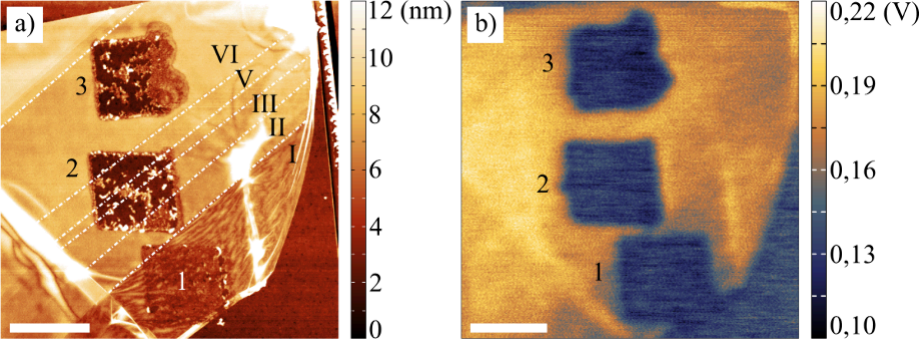
Structures prepared on graphene layers of different thicknesses—the Roman numerals indicate the number of graphene layers, and white lines separate areas of a different number of layers: (a) AFM topography and (b) KPFM surface potential (taken 7 days after oxidation). The scale bar represents 1 μm.

Topography data (Figure 3a) shows that for the monolayer and bilayer, only slight topography changes (e.g., smoothing out surface corrugations typical for exfoliated and transferred graphene) occur. However, more profound changes can be seen on a thicker, multilayered graphene. The LAO procedure led to a bumpy surface with a partially unveiled SiO_2_ substrate and sputtered pieces of torn graphene.

The KPFM image of these structures is depicted in Figure 3b. During the first days after oxidation, apparent surface potential differences between the LAO processed and unprocessed areas are visible. Then, these differences were slowly decreasing (see Section 4.2), which can be fit by the exponential time decay as follows:

$$\Delta V_{\mathrm{SP}}(t)=V_{0}e^{-t/\tau }$$ 1

where Δ*V*_SP_(*t*) is the time-dependent surface potential difference between the structure and the surrounding pristine graphene, *t* is the time in days, *V*_0_ is the initial potential difference, and τ is the decay time constant.

The Raman spectroscopy (with spatial resolution of 200 nm) also indicates the changes associated with the LAO process (Figures 4 and 5). A distinct increase in the D peak intensity (connected with defects, dopants, and bounded molecules) was observed on all three structures. The remaining two peaks (G, 2D) have decreased in intensity within the processed areas, corresponding to the removal or oxidation of graphene.

**Figure 4:**
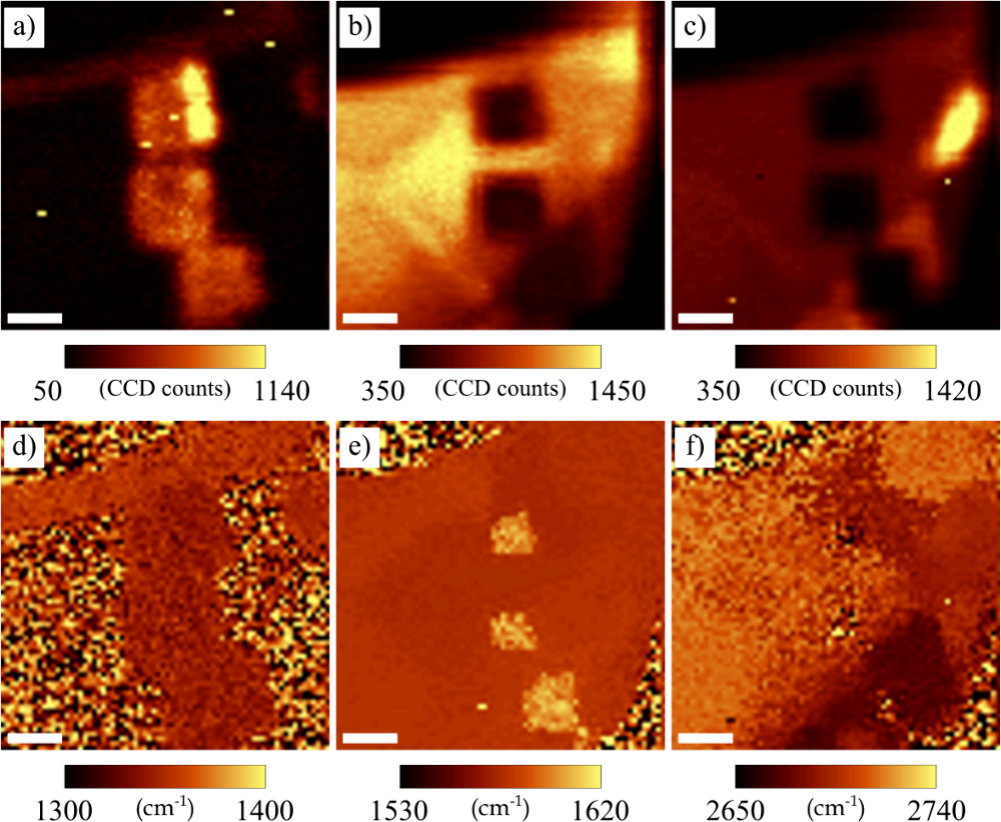
Raman spectroscopy on LAO prepared structures and their surrounding: (a) D peak (∼1350 cm^–1^) intensity, (b) G peak (∼1580 cm^–1^) intensity, (c) 2D peak (∼2700 cm^–1^) intensity, (d) D peak position, (e) G peak position, and (f) 2D peak position. The scale bar represents 1 μm.

**Figure 5:**
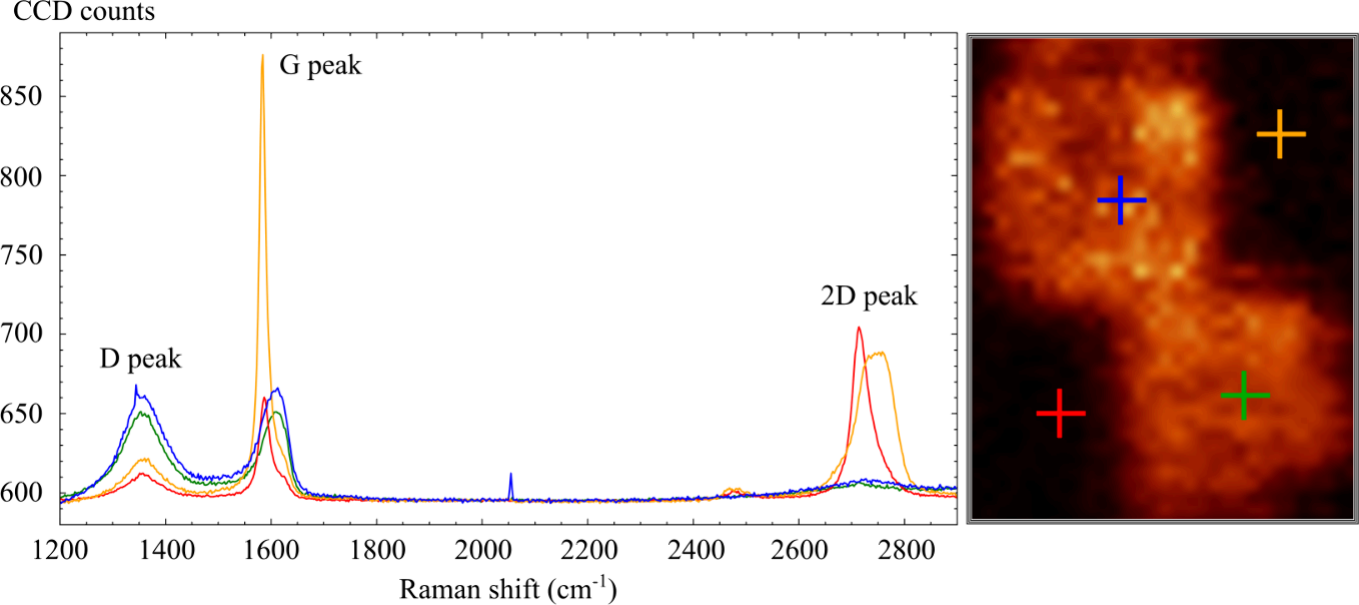
Examples of the Raman spectra: LAO processed graphene—structure 1 (green), pristine graphene (red), LAO processed graphene—structure 2 (blue), and pristine multilayer (orange).

Further indicators of the modified graphene by LAO might be shifts of the prominent graphene peaks. Indeed, as can be seen in Figures 4e and 5, the G peak blueshifted (i.e., to higher energies) for all thicknesses, while its intensity decreased. On the other hand, the D and 2D peaks do not reveal any observable shifts – see Figures 4d,f and 5. However, the intensity, shape and position of the 2D peak of pristine graphene differ depending on the layer number. The noise in the maps (Figure 4d–f) is related to the absence of significant peaks – nearly no 2D peak is present in the centers of the oxidized areas.

### 3.2 Load Force and Substrate

Figure 6 illustrates the range of structures prepared through the LAO processing of graphene under various conditions (i.e., loading forces and substrates). Their characteristics are summarized in Table 1. In the case of the 8.3 nN pulling force and gold substrate (Figure 6a), a significant volume increase occurred, with the graphene layers being lifted by 100 nanometers. Additionally, the KPFM measurements exhibited a significant initial potential difference of about 300 mV relative to the surrounding pristine graphene, which then slowly decreased with time. This rare, specific, and hardly reproducible structure is called a “graphene tent” in the paper. It has been prepared only on graphene placed on the gold electrode, using a high pulling force that acts on the graphene via a water meniscus.

**Figure 6:**
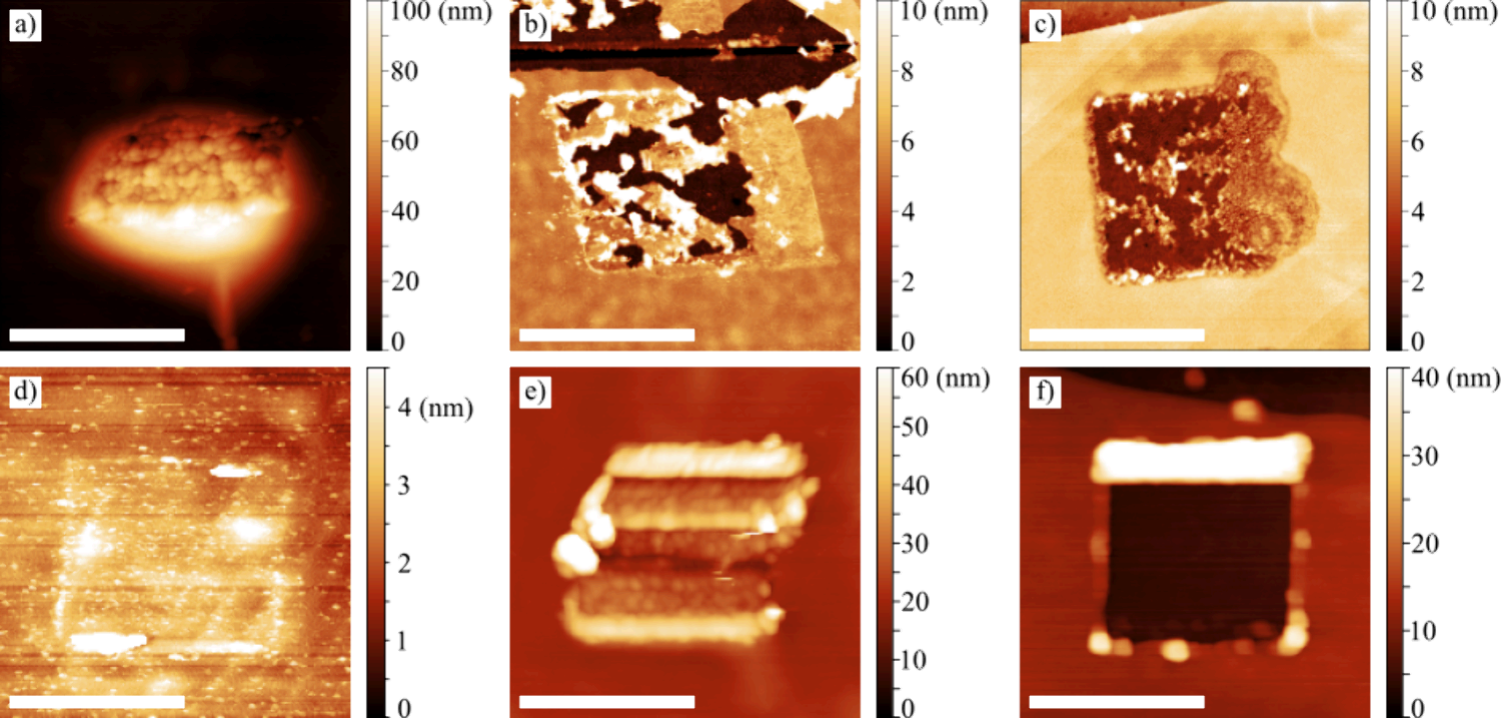
Structures prepared by LAO under different loading forces on two distinct substrates underneath graphene. (a) “Graphene tent” (−8.3 nN, pulling force, gold), (b) partly removed graphene (−7.7 nN, pulling force, silicon dioxide), (c) partly removed graphene (−6 nN, pulling force, silicon dioxide), (d) “nearly invisible square” (−3.2 nN, pulling force, gold), (e) “highly disordered square” (186 nN, pushing force, gold), and (f) hole (279 nN, pushing force, silicon dioxide). The scale bar represents 1 μm.

**Table 1:** Characteristics of the Prepared Structures

type of the structure	applied load force (nN)	substrate	initial potential difference (mV)	time decay constant τ (days)
“graphene tent”	–8 to −9	Gold	300	10
partly removed graphene	–6 to −8	SiO_2_	200–300	20
“nearly invisible square”	–3 to 18	Gold	15–50	1.5–10
“highly disordered square”	100 to 300	Gold	200	25–33
hole	200 to 300	SiO_2_	120	33–50

The results obtained for the pulling force of −7.7 nN, and the silicon dioxide substrate underneath graphene are shown in Figure 6b. For completeness, it is worth mentioning that the tip–sample voltage was −7 V instead of the typical −9 V. In Figure 6b,c, the areas with the partially removed graphene are clearly visible. The remaining graphene has been turned into “bumps”, scattered across the square. These residues still show Raman graphene peaks, with the D peak indicating disorders. Similarly to the previous case, the initial potential differences measured by KPFM were also high, reaching 200–300 mV. However, the discharging process was even slower.

The low absolute values of the loading force relate to a less observable patterning effect resulting in “nearly invisible squares” as demonstrated in Figure 6d (pulling force −3.2 nN). Their subtle character makes them nearly unobservable in the AFM topography image. KPFM was able to distinguish the structures due to their different surface potential. However, the potential difference rapidly vanished with time (the time decay constant τ reached a value as small as 1.7 days). The Raman spectroscopy did not detect changes (the D peak was absent).

Furthermore, a stiffer DCP20 probe was used to apply higher loading forces. The LAO process carried out at a pulling force of 186 nN deformed the graphene into shapes resembling “walls” and “trenches” (see Figure 6e), while the process performed at a significantly higher force of 279 nN resulted in the complete removal of graphene layers, creating a squared hole. At the same time, a kind of high “wall” appeared on the “rear” side (where the tip motion ended), likely formed by the graphene scratched away from the rest of the square. Unsurprisingly, the square showed no graphene peaks in the Raman spectroscopy; only silicon peaks were detected.

The initial potential difference between the “highly disordered squares” (Figure 6e) and the ambient graphene was approximately 200 mV and decreased very slowly with a time decay constant τ of 25 to 33 days. The potential difference associated with the holes (Figure 6f) started at about 120 mV and discharged with a decay time constant τ of 30 to 50 days. Here, the charge was probably accumulated at the insulating silicon dioxide surface.

### 3.3 Simulation Results

Electric potential and electric field were calculated in COMSOL Multiphysics (see Section 2) for the parameters as follows: *R* = 100 nm (corresponding to the real radius of the tip), *h* = 1 nm, RH = 67.5%, *V* = −10 V. The Kelvin radii of the meniscus were stated as *r*_1_ = 16.3 nm, *r*_2_ = 1.27 nm (details of the model in Supporting Information).

Figure 7 illustrates the electric potential for 1, 2, 4, and 8 graphene layers, with intercalated layers containing air. As the number of layers increases, the potential drop occurs over a longer distance, and the electric field between the tip and the substrate becomes weaker. Figure 8 shows the electric field profiles: the green profiles are calculated for air beneath the graphene layers, while the blue profiles pertain to water beneath the layers. Water notably reduces the electric field in the bottommost gap, whereas in the other gaps, the field is marginally stronger. The strength of the electric field within the graphene layers remains nearly unaffected by the presence of water, particularly for four or more layers. However, the most significant finding is that the electric field in both the graphene and the intercalated air layers diminishes as the number of layers increases.

**Figure 7:**
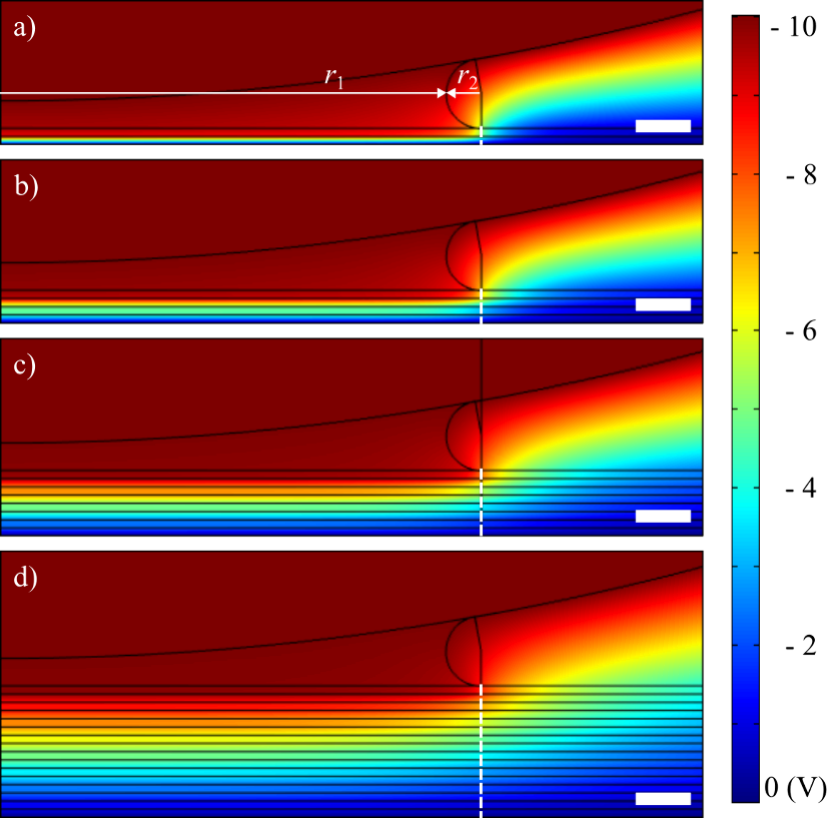
COMSOL Multiphysics simulation of electric potential (*V*) between the AFM tip and (a) the graphene monolayer, (b) the bilayer, (c) four layers, and (d) eight layers with a water meniscus. The field profiles along the white line are shown in Figure 8. The scale bar represents 2 nm.

**Figure 8:**
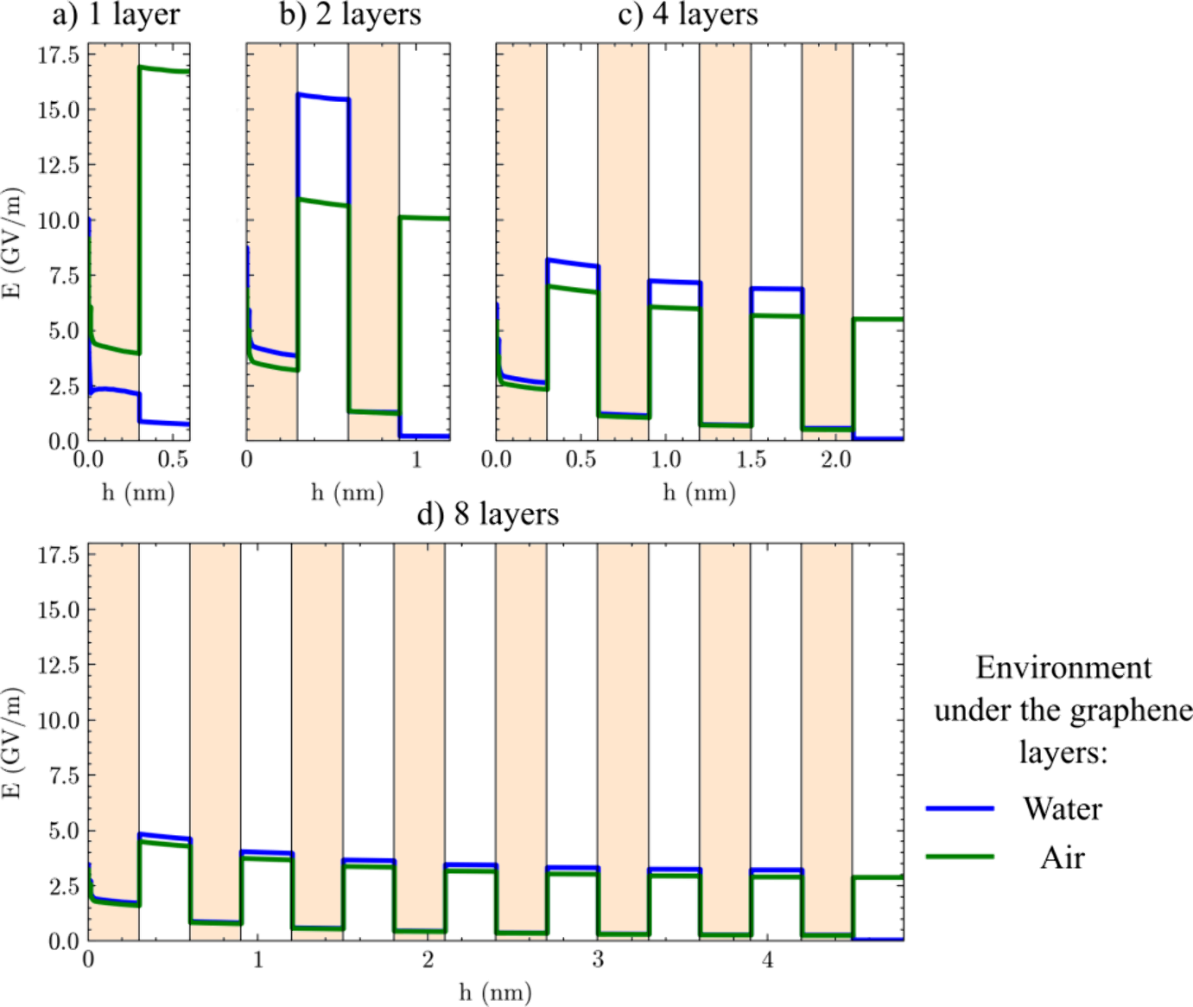
Profiles of the electric field (*E*) between the tip and the substrate for different numbers of graphene layers: (a) monolayer, (b) bilayer, (c) four layers, (d) eight layers, with water (blue) or air (green) under the graphene layers. The profiles are calculated along the white lines in Figure 7. The electric field intensity generally weakens as the number of layers increases.

## 4 Discussion

### 4.1 Number of Layers and Substrate

Conducted experiments imply a strong dependence of the LAO processing on the number of layers. While oxidation may occur on a single graphene layer, in the case of three or more layers, graphene is generally removed from the larger parts of the processed area. This agrees with the results of former studies aimed at measuring adhesion forces between graphene and its substrate. For silicon dioxide, a clear difference between the single layer and the thicker layers of graphene was found. The adhesion of the single layer reaches a value of about 0.45 J/m^2^, whereas the bilayer, trilayer, and multilayer display values of about 0.31 J/m^2^.^22^ These data were measured using the pressurized blister test method and were supported by theoretical calculations, reaching 0.45 J/m^2^ (single layer) and 0.37 J/m^2^ (bilayer) for the corrugated substrate.^22,23^ A flat substrate would lead to a lower difference.^23^

Our results qualitatively agree with this previous work. We can remove a large part of weakly bonded multilayer graphene while the single-layer graphene remains in its place and undergoes an oxidation process.

The adhesion of graphene also depends on the type of substrate. Several experiments and theoretical calculations in previous studies indicate a stronger bonding with less-conductive materials.^21,24,25^ The value of adhesion energy was determined as 0.27 J/m^2^ for silicon dioxide and 0.255 J/m^2^ for gold.^24^ Although this difference is not significant, it qualitatively supports the conclusion that graphene binding on metals is weaker than on oxides.^22,23^

On the gold electrode, we have observed a great lift of layers (“building the graphene tent”, Figure 6a). However, on the silicon dioxide surface, graphene has been lifted only at some points, creating “bubbles”, which subsequently burst and collapsed on the sides. Consequently, the bumps of defective graphene remain between the former “bubbles”. The larger extent of removed graphene area in Figure 6c than in Figure 6b may be related to a higher oxidizing voltage (−9 V compared to −7 V). However, the “tent” did not appear in these cases. It implies stronger adhesion to the substrate, which corresponds to the results found in the literature.

While the adhesion weakens as the number of layers increases, the work function grows with this number, especially between 1 and 10 layers.^26,27^ The calculated difference in the work function between 1 and 10 layers was reported as 60 meV^26^ and 400 meV,^27^ respectively. A higher work function makes graphene oxidation more difficult, especially when the adhesion force decreases simultaneously. For all graphene layers, the value of the work function varies around 4.5 eV.^26,27^ It coincides with the value of the oxidation threshold voltage, which was experimentally determined as 4.5 V^3^ and between 3.8 and 4.2 V, respectively.^10^

COMSOL calculations have revealed that the electric intensity is decreasing with an increasing number of layers. This effect, together with an increasing work function, makes the oxidation of graphene multilayers less probable. A wide range of structures in Figure 6 did not experience any oxidation, only mechanical modification. This is discussed further in Section 4.3.

### 4.2 Surface Potential

The evolution of surface potential difference between LAO-processed structures and ambient graphene, measured regularly within forty days, is depicted in Figure 9. The time evolution of potential difference can be divided into two regions: the discharging region and the stability region. The former one lasted for about 20 days. In this time range, the potential differences for all structures were decreasing rapidly, so we decided to fit them with an exponential function (eq 1), which is the solution of the differential equation of a capacitor (capacitance *C*) discharging through a resistor (resistance *R*):

$$R\frac{dQ}{dt}+\frac{Q}{C}=0$$ 2

where *Q* is the total amount of charge in the structure, and τ = 1/*RC* represents the decay time constant. This simple model is sufficient for fitting the data if the surface potential is directly proportional to the charge delivered during the LAO process. Because of this model, the first region is called the “discharging region”. The LAO was carried out in a high-humidity atmosphere; nonetheless, afterward, the sample was kept mainly in drier conditions (relative humidity varied between 20 and 40%), preventing faster charge displacement.

**Figure 9:**
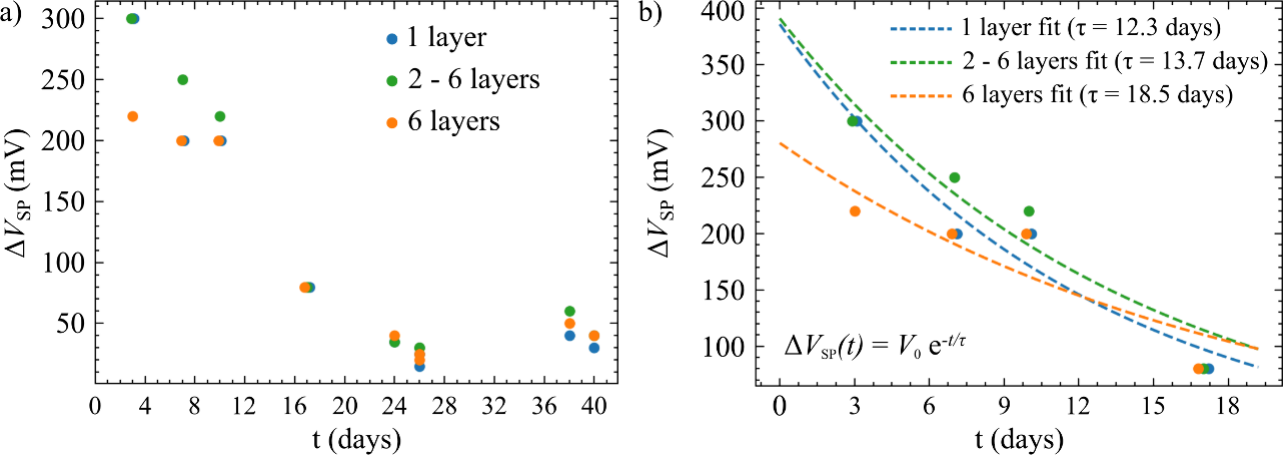
Time evolution of surface potential difference (Δ*V*_SP_) between LAO-processed structures and ambient graphene. The points represent the measured data; the dashed curves represent the exponential fits. (a) The evolution, including the discharging region (first 20 days) and the stability region. (b) Discharging region with exponential fits.

However, after about 20 days, the charge displacement was finished. In the following days, the potential difference stayed unchanged. Its values were for structures 1, 2 and 3, 29 ± 9, 39 ± 11 and 35 ± 11 mV, respectively. The difference was smaller for the single layer (structure 1) than for the others. The existence of the stability region implies a permanent local transformation of the graphene structures, due to the destruction of thicker layers and oxidation of the monolayer. Here, the potential difference can indicate the presence of bonded oxygen groups.

The discharging rate (represented by the decay time constant τ) depends on the result of the LAO process – see Table 1. The “nearly invisible squares” (Figure 6d) underwent no visible deformation, and the corresponding time decay constant τ reaches 1.5–10 days. On the other hand, the strongly deformed graphene layers (Figure 6e) lose their charge with τ ranging from 25 to 33 days. The holes exhibit even slower discharging (τ= 33–50 days) because of the charge forced to diffuse through an insulating silicon dioxide layer.

These results correspond to the theory of electrical resistivity in defective metals, which states that an increase in the density of defects decreases conductivity.^28^ In graphene (treated as a metal due to its zero-band gap), deformed by the LAO processing, the higher concentration of defects decreases the charge flow, leading thus to a higher value of τ*.*

### 4.3 Loading Force

As stated before, the LAO processing of graphene strongly depends on the substrate. The graphene layers tend to tear themselves rather than fully peel off from the silicon dioxide. As a result, we can get structures like Figure 6b or c. However, to peel off the graphene from the entire area (i.e., to create a “graphene tent” (Figure 6a)) is impossible due to the strong adhesion of graphene to SiO_2_. A hole (Figure 6f) can also be prepared if the graphene is scratched away by a tip acting with a load force of 200–300 nN.

However, in the case of graphene on the gold electrode, one can observe more variations. No holes were observed on this substrate despite using pulling forces of hundreds of nanonewtons. Graphene was deformed to “walls and trenches” like in Figure 6e. The smaller force would create “nearly invisible squares” (Figure 6d), probably only charged (no D peak in Raman spectra). They were observed for a force ranging from 20 nN in pushing mode to 7 nN in the pulling mode. For a pulling force of about 8 nN, the “tents” (Figure 6a) were sometimes created. The space under them could be filled with water, obtained from the intercalated water layer.^15–20^

## 5 Conclusions

Local anodic oxidation of graphene layers can prepare a wide range of structures whose characteristics depend on relative humidity, applied voltage, AFM tip shape, and scanning speed (i.e., the time of oxidation). The article focused on other parameters whose precise control is essential for achieving reproducible LAO results: number of graphene layers, loading force, and adhesion of graphene to the substrate.

The reproducible preparation of graphene oxide was achieved on a monolayer firmly adhered to the silicon dioxide surface at a pulling force between the AFM tip and graphene. The pulling (not pushing) force is necessary to prevent the mechanical removal or destruction of graphene. At such a pulling force, the contact between the AFM tip and graphene is mediated via a water meniscus, a source of O- and OH- groups for oxidation. At the same time, it is necessary to ensure that the pulling force does not withdraw the graphene from the substrate, resulting in instability in the oxidation process. For this purpose, the strong attachment of a graphene monolayer on the silicon dioxide substrate proved to be sufficient, unlike the weak bonding of graphene multilayers or graphene placed onto a gold substrate.

## Supplementary Material



## Data Availability

The data that support the findings of this study are at https://zenodo.org/records/17939734.

## Notes

Additional information concerning the experimental setup; the calculation of the approximate shape of the water meniscus (PDF)
