# Supplementary material for: Local Anodic Oxidation of Graphene: The Role of Number of Layers, Load Force, and Substrate
Source: ACS Omega. 2026 Jan 22;11(4):6434–41. doi: 10.1021/acsomega.5c10137 (PMC12878732; doi:10.1021/acsomega.5c10137)
Supplement: Supplementary file 1 [file ao5c10137_si_001.pdf]

Supporting information: Local anodic oxidation of graphene: The role of number of layers, load force and substrate

Jan Vymazal [a, b], Miroslav Bartošík [a, b, c] *, Martin Konečný [a, b], Jakub Piastek [a, b], Jindřich Mach [a, b], Linda Supalová [a, b], Ondřej Špaček [a, b] and Tomáš Šikola [a, b]

(a) *Central European Institute of Technology – Brno University of Technology (CEITEC BUT) Purkyňova 123, 612 00 Brno, Czech Republic*

(b) *Institute of Physical Engineering, Brno University of Technology, Technická 2, 616 69 Brno, Czech Republic*

(c) *Department of Physics and Materials Engineering, Faculty of Technology, Tomas Bata University in Zlín, Vavrečkova 5669, 760 01 Zlín, Czech Republic*

* bartosik@fme.vutbr.cz

In this section of supporting information, the experimental setup is pictured. The exfoliated graphene layer (Fig.1a, b, c) was placed over the gold electrode and the silicon dioxide substrate. The whole experimental platform (equipped with gold electrodes on a 285 nm thick non-conductive layer of silicon dioxide (SiO_2), thermally grown on a silicon substrate) is pictured in Fig.S1a. The details of the electrodes are shown in Fig.S1b. The broader edges of the electrodes were connected via a silver paste. The sample was then grounded through a copper wire plugged into the microscope (see Fig.S1c).

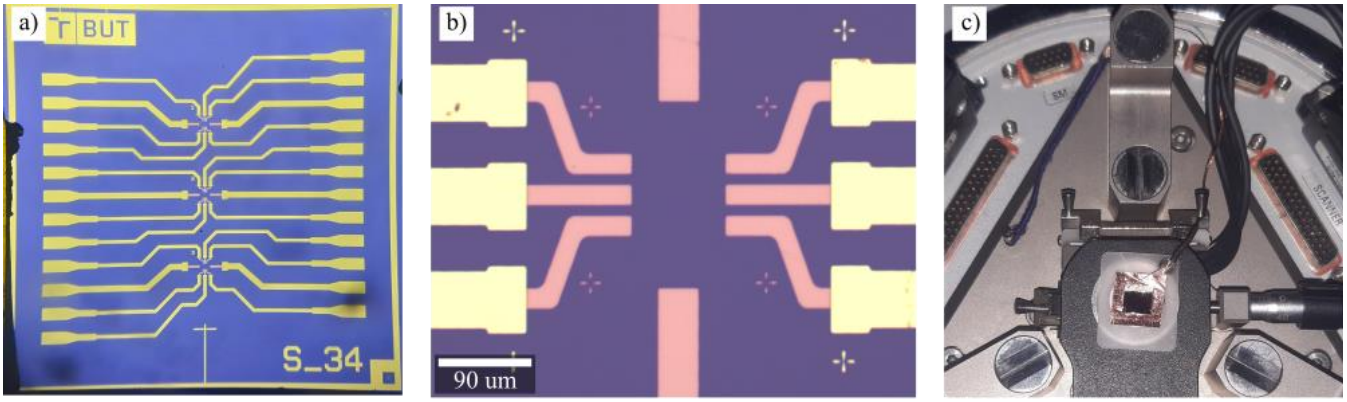

Figure S1: a) The platform for graphene oxidation – Si substrate covered by a silicon dioxide layer with gold electrodes on the top. b) Detail of the electrodes. c) The platform placed on the AFM stage was grounded via a silver paste and copper wire.

Shape of the meniscus

To understand the water meniscus and calculations of electromagnetic fields between the AFM tip and graphene, the conditions of the meniscus creation should be explained first. The independent variables are the relative humidity of the air (*RH*), the tip-sample distance (*h*), and the radius of the tip (*R*). The geometry of this problem is depicted in Fig.S2. The AFM tip has a conic shape, with its apex approximated by a sphere of radius *R*, located at a distance *h* from the planar hydrophilic sample. In this approximation, both the sample and tip are perfectly wettable. The water meniscus is featured as a column between the tip and the sample. Its smallest radius is labelled r_1 [29] (positive value), while the external curvature of the water surface is labelled r_2 (negative value). Thanks to the perfect wettability, the water surface is curving concavely.

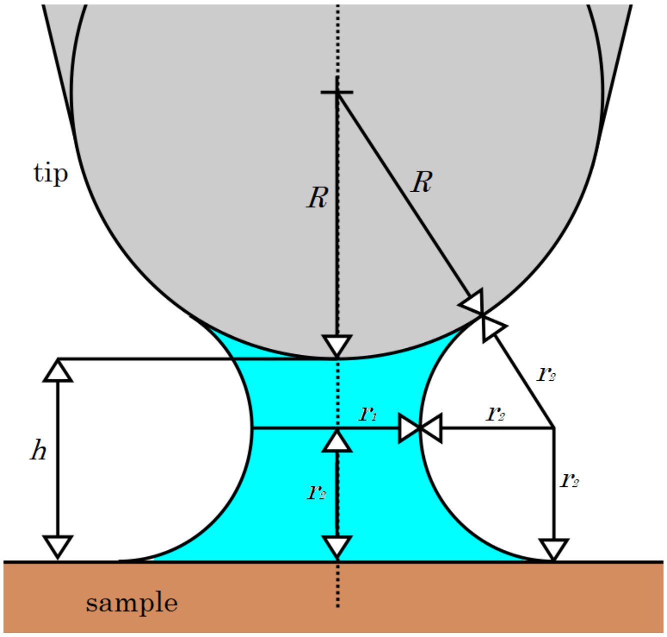

Figure S2: The geometry of the water meniscus between the AFM tip and the sample.

This mathematical model, taken from [29, 30], enables calculating an approximate shape of the water meniscus, which can be compared with experimental results and used for calculating the electric intensity. Geometric and thermodynamic conditions determine the meniscus shape [29]. The thermodynamic condition, based on the Kelvin equation, is generally valid. On the other hand, the geometric condition based on the Pythagoras formula can be used only in a simplified model of perfect wettability. In this condition, a triangle can be imagined among the centre of the sphere, approximating the tip, the centre of external curvature of the meniscus, and the corresponding point on the axis of symmetry (marked in Fig.S2). In this case, the Pythagoras formula has the form of:

$$(R + r_2)^2 = (R + h - r_2)^2 + (r_1 + r_2)^2$$

(S1)

The r_1 and r_2 radii are also present in the Kelvin equation, which connects them with the temperature and relative humidity of the environment [29]:

$$r_K = \left(\frac{1}{r_1} + \frac{1}{r_2}\right)^{-1} = \frac{\gamma_{LV} V_m}{RT \ln\left(\frac{p_V}{p_V^*}\right)},$$

(S2)

where r_K is the Kelvin radius, γ_{LV} is the interface energy of water and water vapor, V_m is the molar volume of the water, R is the molar gas constant, T is the thermodynamic temperature, p_V is the water vapor pressure and p_V^* is the saturated water vapor pressure. The ratio of these values (p_V/p_V^*) represents the relative humidity RH. At room temperature we approximately get $\frac{\gamma_{LV} V_m}{RT}$ = 0.54 nm [29], so the r_1, r_2 radii are connected through the relation:

$$\left(\frac{1}{r_1} + \frac{1}{r_2}\right)^{-1} = r_K(RH) = \frac{0.54}{\ln RH}.$$

(S3)

The equations (S1) and (S3) form a set of equations with two variables. Expressing the r_2 with the remaining values in both equations leads to relations:

$$r_2 = \frac{r_1 r_K}{r_1 - r_K} \qquad r_2 = (2R + h - r_1) \pm \sqrt{(2R + h)\left(\frac{R - r_1}{2}\right)}.$$

(S4)

The expression before the root is principally positive, but $r_2 < 0$ (see higher). Because of that, the expression with + can be excluded. As a result (after alterations), the following equation is obtained:

$$r_1^2 - (2R + h + 2r_K)r_1 + (2R + h)r_K - 2\sqrt{(2R + h)\left(\frac{R - r_1}{2}\right)}(r_K - r_1) = 0.$$

(S5)

It was resolved using numerical optimization methods, finding the appropriate value of r_1. From it, the value of r_2 was also calculated.
